# Applications of Carbon Dots for the Photocatalytic and Electrocatalytic Reduction of CO_2_

**DOI:** 10.3390/molecules27031081

**Published:** 2022-02-06

**Authors:** Beatriu Domingo-Tafalla, Eugenia Martínez-Ferrero, Federico Franco, Emilio Palomares-Gil

**Affiliations:** 1 Institute of Chemical Research of Catalonia, The Barcelona Institute of Science and Technology (ICIQ-BIST), Avda. Països Catalans, 16, E-43007 Tarragona, Spain; bdomingo@iciq.es (B.D.-T.); emartinez@iciq.es (E.M.-F.); 2 Departament d’Enginyeria Electrònica, Elèctrica i Automàtica, Universitat Rovira i Virgili, Avda. Països Catalans, 26, E-43007 Tarragona, Spain; 3 ICREA, Passeig Lluís Companys 23, E08010 Barcelona, Spain

**Keywords:** carbon dots, graphene quantum dots, photocatalysis, electrocatalysis, CO_2_ reduction, single atom, metal free

## Abstract

The photocatalytic and electrocatalytic conversion of CO_2_ has the potential to provide valuable products, such as chemicals or fuels of interest, at low cost while maintaining a circular carbon cycle. In this context, carbon dots possess optical and electrochemical properties that make them suitable candidates to participate in the reaction, either as a single component or forming part of more elaborate catalytic systems. In this review, we describe several strategies where the carbon dots participate, both with amorphous and graphitic structures, in the photocatalysis or electrochemical catalysis of CO_2_ to provide different carbon-containing products of interest. The role of the carbon dots is analyzed as a function of their redox and light absorption characteristics and their complementarity with other known catalytic systems. Moreover, detailed information about synthetic procedures is also reviewed.

## 1. Introduction

Anthropological CO_2_ emissions are the primary driver of global climate change, since their growth from mid-20th century has actually reached 36 billion tons per year [1]. As a way to contribute to climate change mitigation, CO_2_ capture and utilization has started to attract attention worldwide because it can turn waste CO_2_ emissions into valuable products, such as chemicals and fuels [2], contributing to a sustainable economy with a closed carbon cycle.

The catalytic CO_2_ reduction reaction (CO_2_RR) driven by light (photocatalysis) or electricity (electrocatalysis) powered by renewable sources are promising approaches to replicate nature’s process of CO_2_ conversion into chemical energy [3]. They are extremely attractive ways of using CO_2_ as a feedstock for the production of fuels or value-added monocarbon (C_1_) and multicarbon (C_≥2_) chemicals, such as formic acid/formate (HCOOH/HCOO^−^), carbon monoxide (CO), methane (CH_4_), ethylene (C_2_H_4_), methanol (CH_3_OH), or ethanol (C_2_H_5_OH). Due to the wide variety of carbonaceous products that can be potentially formed, as well as the competitive side Hydrogen Evolution Reaction (HER) which typically lowers the efficiency of the overall CO_2_RR process in aqueous media, the selectivity toward the formation of a specific target product is the major challenge for achieving an efficient CO_2_RR. Furthermore, the extreme inertness and stability of the CO_2_ molecule pose both thermodynamic and kinetic obstacles to an efficient CO_2_ activation and conversion [4]. Several transition metal-based catalysts have been reported so far for efficient electro- and photocatalytic CO_2_RR, including homogeneous and heterogenized molecular catalysts, reticular 2D/3D materials (e.g., metal−organic frameworks (MOFs), covalent-organic frameworks (COFs)), single atom catalysts and nanostructured heterogeneous materials based on metal or metal-oxide surfaces [5,6,7,8,9]. However, the performances of the state-of-the-art systems developed so far need to be further improved to fulfill the applicative and technological requirements in terms of cost, selectivity and durability.

Carbonaceous materials, including carbon nanotubes (CNT), graphite, graphene and porous carbon, are known to possess good chemical and mechanical stability, excellent electrical conductivity and tunable electrical structure, representing ideal components for energy conversion devices [10,11,12]. In the past few years, various carbon-based nanomaterials have been successfully employed for an efficient catalytic CO_2_ reduction, either in combination of metal/metal oxide-based catalysts or as metal-free catalysts themselves [13,14,15,16]. They have even been reported for the obtention of C_≥2_ products [13,17] on account of their capability to decrease the energy barrier of the C−C coupling [18]. An extensive variety of metal-free carbon based catalysts for CO_2_RR have been reviewed elsewhere [10].

**Scheme 1:**
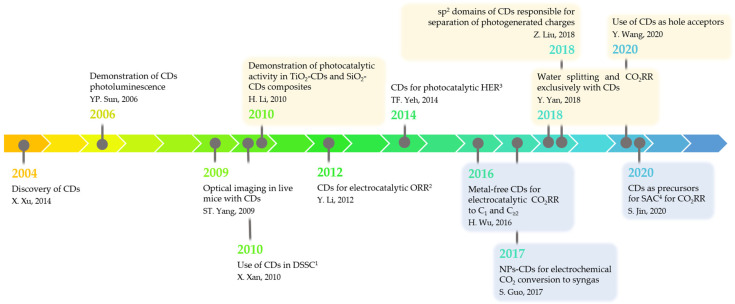
Timeline with the most relevant applications of CDs in photocatalysis (yellow) and electrocatalysis (blue) for CO_2_RR and in other fields. ^1^ DSSC: dye sensitized solar cells, ^2^ ORR: oxygen reduction reaction, ^3^ HER: hydrogen evolution reaction, ^4^ SAC: single atom catalysts [30,31,29,28,50,21,20,17,52,45,47,90,48].

Carbon dots (CDs) are a new kind of nanocarbon material and have been gaining attention as promising candidates for the design of novel materials for photocatalytic and electrocatalytic applications [19,20,21,22,23,24,25], along with numerous applications in other fields [26,27,28], since they were first discovered in 2004 [29] (see Scheme 1). CDs are surface-functionalized graphitic or amorphous carbon nanoparticles (size below 10 nm) with high chemical stability, excellent photo/electro properties, electron transfers/transport capacities and easy large-scale production [30,31]. To date, they have been thoroughly characterized with several techniques, and great progress has been made on tuning their properties by changing the synthetic parameters. In this regard, numerous heteroatom doping strategies have been developed in order to improve their fluorescence properties, i.e., to increase their quantum yields and tune the wavelength emission. However, heteroatom doping also breaks the electroneutrality and provides CDs with a highly improved electronic structure and enhanced catalytic properties. Moreover, the optical properties of CDs have been associated to photoinduced redox characteristics, since the photoexcitation results in desirable charge separation and transfer for photocatalytic applications [32]. Finally, CDs can be doped with Earth abundant metals such as Fe, Co, and Mn, which, in combination with the heteroatoms (H), could form M-H-C centres which are reported to act as catalytic active sites [33,34].

This review aims to provide a general overview about the applications of CDs for photocatalytic and electrocatalytic CO_2_RR, critically assessing the role of the CDs in the reported catalytic systems. In the next section, the main methods recently proposed for the synthesis, purification, and characterization of the CDs will be presented, as well as the most commonly used strategies for CDs doping and hybridization with metal/metal-oxide materials. The applications of CDs for photocatalytic and electrocatalytic CO_2_RR will be discussed in the Section 3 and Section 4, respectively, systematically comparing the metal-free CDs catalysts with the metal composite materials. Based on the data herein presented, the role of CDs in the catalytic CO_2_RR process will be discussed, providing some hints on future perspectives of the research field.

## 2. Strategies for CDs Synthesis

### 2.1. Synthesis Methods

**Figure 1:**
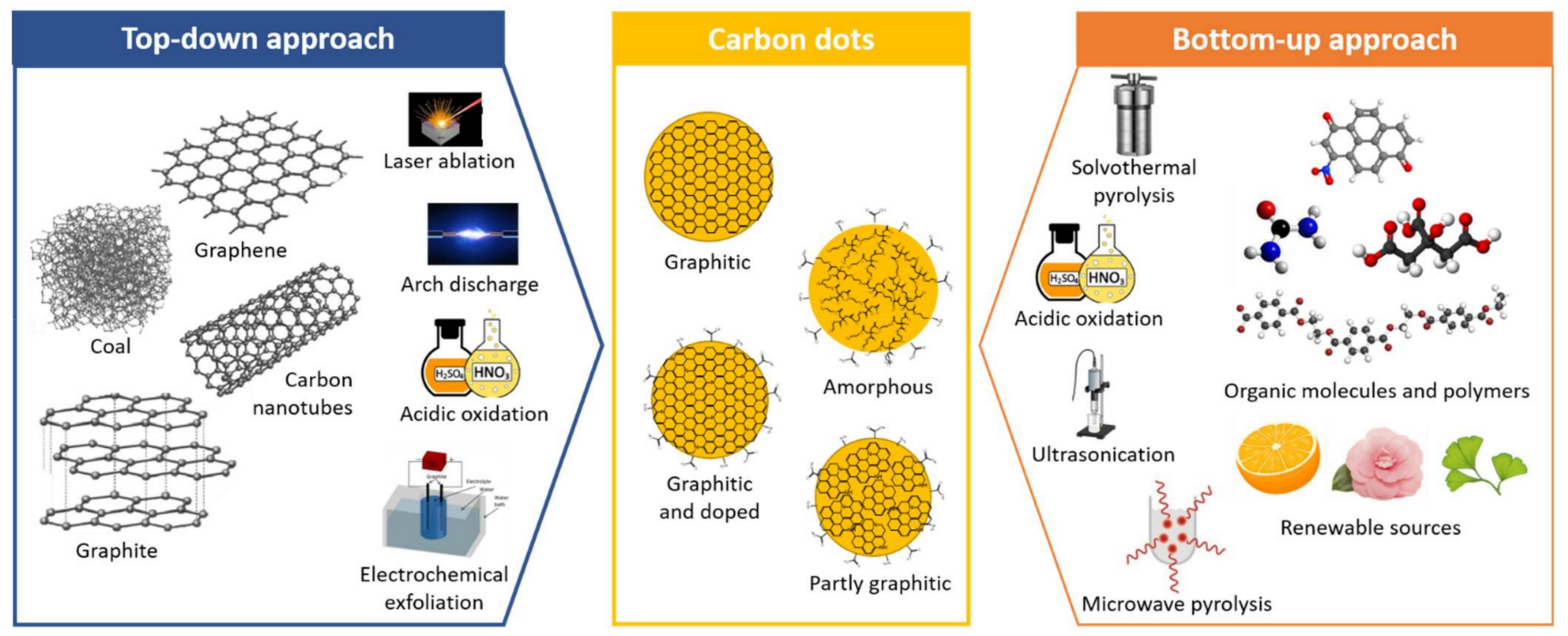
Schematic description of the different approaches followed for the synthesis of carbon dots.

The preparation of CDs can be described by two main synthetic methodologies: the top–down approach, which consists in the breaking of bulk carbon structures, and the bottom–up approach, which refers to the formation of nanoparticles from molecular precursors via chemical reactions (Figure 1). By tuning the reaction precursors and synthetic procedures, a vast number of CDs and heteroatom-doped CDs with distinct properties and applications can be obtained. Indeed, a comparison between top–down and bottom–up approaches showed that CDs and N-doped CDs obtained from these two methods have differentiated photoluminescence properties and chemical states. For instance, both types of CDs may feature varying distributions of functional groups (i.e., hydroxyl, carbonyl and carboxyl, amine, and pyridinic and pyrrolic N), and notable changes in sp^3^-carbon content depending on the specific synthetic conditions [35].

#### 2.1.1. Top–Down Approach

Top–down methods comprise physical and chemical strategies to break the carbon structures. This approach usually results in dots with high crystallinity and intact structures, most commonly named graphene quantum dots (GQDs) because of the typical graphitic organization of the carbon atoms in the nanoparticle. Arc-discharge was the first top–down method to isolate GQDs during the synthesis and purification of Single-wall carbon nanotubes (CNTs) [29]. This technique consists of breaking big-size carbon compounds with an electrical arc-discharge, while their post-synthetic acid oxidation enables to obtain surface passivated, stable and heteroatom doped GQDs. Alternatively, GDQs can be obtained by laser ablation, which consists of removing material from a solid surface by irradiating with a laser beam [36]. Carbon sources that have been employed to obtain GQDs following this method include graphite immersed in polyethyleneimine (PEI) and ethylenediamine (EDA) [37], activated carbon suspended in n-heptane [38], or solid carbon immersed in acetone [39]. Moreover, acidic oxidation is the treatment of graphene oxide, soot, coal, carbon black, or activated carbon with an oxidizing agent at temperatures of 60–100 °C, which exposes the edge sites from which the carbon material is exfoliated. For example, GQDs have been obtained from Multi-Walled CNTs oxidized with a mixture of HNO_3_/H_2_SO_4_ [40,41,42], from carbonized coal treated with HNO_3_ [43], from anthracite coal treated with H_2_SO_4_ and HNO_3_ [44] and from graphene oxide treated with periodic acid, H_2_SO_4_ or HCl [17,45,46,47]. Furthermore, the electrochemical exfoliation of carbon-based materials revealed to be an effective synthetic method to obtain GDQs. It consists in applying a static potential between two electrodes acting as the carbon sources, including CNTs [48], graphite rods [49,50,51,52], and reduced graphene oxide films [53]. Furthermore, GQDs have been produced via electrochemical exfoliation in large scale by applying a constant voltage of 15 V between a pre-baked carbon anode and stainless steel (cathode) [54], or from two clean carbon rods placed in parallel in deionised water, with static potential of 15–60 V [55]. Finally, other top–down techniques following physical routes can be found in the literature, such as microwave (MW) irradiation [56] and ultrasounds [57,58], but they are less common.

Moreover, GQDs usually require heteroatom doping, mostly with nitrogen, to enhance their photoluminescent or catalytic properties. Top–down synthesized GQDs have been N-doped by thermal treatment with ammonia solution [52,55] or in situ with solvents, such as dimethylformamide (DMF) [17,59], which decompose at high temperatures forming ammonia and other compounds.

#### 2.1.2. Bottom–Up Approach

Bottom–up methods tend to yield CDs with an amorphous carbon core and abundant doping sites and surface functional groups. The most common synthetic route is the hydrothermal/solvothermal method, based on a thermal decomposition of organic precursors in aqueous or organic solution at temperatures typically in a range between 100 and 250 °C. Similar conditions can be achieved with the MW synthesis, which is another common, effective way to synthesize CDs in a shorter reaction time [60]. For both types of synthesis, depending on the temperature or power, reaction time and precursor, the physicochemical properties of the CDs, such as size, crystallinity, solubility, can be controlled. Precursors can be obtained from renewable sources, such as orange juice [61], shrimp egg [62], ginkgo leaves [63], extract of waste tea [64], pine pollen [65], Camellia japonica flowers [66], or waste cotton linter [42]. Other renewable sources are reviewed elsewhere [67]. However, the most conventional sources are commercial precursors, such as citric acid [68,69,70,71,72,73,74], glucose [75,76,77,78], ascorbic acid [79,80], polyethylene glycol [81,82], and ethylene glycol [83]. Other heteroatom-containing precursors, such as urea [68,84,85,86,87,88,89,90] chitosan [91,92,93,94], ethylenediamine [37,71,73,77,95], thiourea [72,96], cysteine [74], cetrimonium bromide (CTAB) [97], phenylenediamine [98], ammonium citrate [99], and nitropyrene [100,101] are used to dope the CDs with Nitrogen and/or Sulphur (N-CDs, S-CDs and N,S-CDs). N-doping can also originate from thermal treatment of the carbon precursor in N-containing solvents such as acetonitrile [90]. 

Another facile approach is ultrasonication, which is the application of intense ultrasound waves into liquids and slurries [102]. For instance, glucose in NaOH aqueous solution has been recurrently use to obtain CDs by ultrasonication [103,104,105,106,107,108,109]. Other approaches for synthesis of CDs have been explored, although they are less common. For instance, acidic oxidation of organic molecules with strong acids at low temperatures (60–100 °C) is an approach with reduced energetic consumption compared to the pyrolytic methods. CDs can be obtained from the oxidation of sucrose [110], ethylene glycol [111], or even pumpkin [112]. Alternative processes are microplasma assisted synthesis at room temperature from o-phenylendiamine [113], thermochemical processing without solvent from N-succinimide [114], citric acid and branched polyethylenimine (BPEI) [115], or only citric acid [116]. Recently, chiral carbon dots have been obtained from cysteine with radical-assisted chemistry at room temperature with Cu(II) as a catalyst [117].

Numerous studies have thoroughly investigated the characteristics of CDs as a function of the nature of the precursors and synthesis conditions. For instance, Miao et al. reported that, by regulating the thermal-pyrolysis temperature and N-doping, the maximum emission of the resulting N-CDs gradually shifts from blue to red through controlling the extent of graphitization and the amount of surface functional groups, -COOH and -NH_2_ [87]. Huan Yuan et al. performed a similar study to demonstrate that surface-passivation of the N-CDs by N-doping can improve the emission efficiency due to the single electron transition resulting from the single functional groups [118]. Khavlyuk et al. found that the CDs appearance from amorphous or well-carbonized spherical particles to onion-like ones is controlled by solvent polarity, whereas the degree of N-doping and the nature of the emissive centers in N-CDs are mostly influenced by the thermal treatment conditions [119]. The systematic study of Crista et al. revealed that both the solvothermal and microwave assisted thermal synthesis of N-CDs from citric acid and urea led to nanoparticles with similar sizes, identical excitation-dependent blue-to-green emission, and similar surface-functionalization. However, it was found that microwave strategy is more efficient towards N-doping than hydrothermal synthesis. Furthermore, microwave routes were found to be more suitable for high-yield synthesis (~27–29%), while hydrothermal synthesis present almost negligible synthesis yields (~2%) [120]. Finally, Sendão et al. conducted a comparative life cycle assessment of solvothermal and microwave-assisted thermal synthesis routes for N-CDs derived from citric acid and urea. They reported that the environmental cost of the electrical consumption during the hydrothermal synthesis advises against the use of this technique. Therefore, the most sustainable option in order to obtain high quantum yield N-CDs is microwave-assisted treatment of an aqueous solution of urea and citric acid [68].

**Figure 2:**
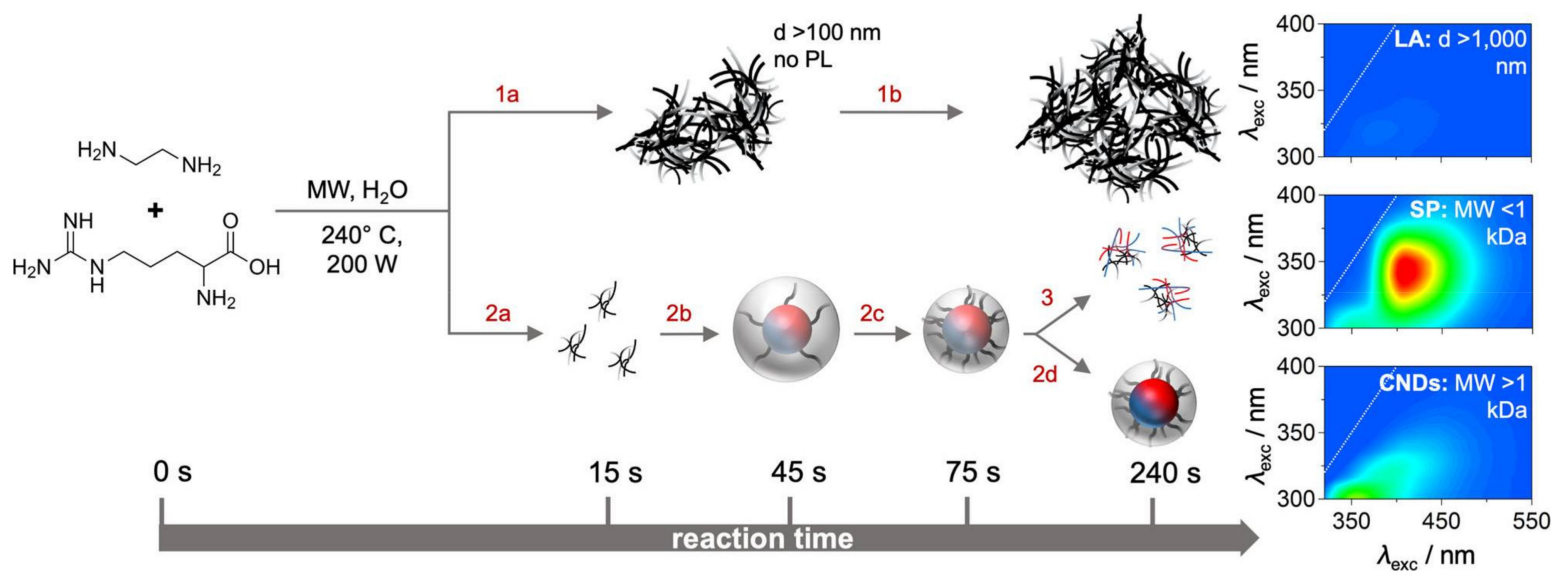
Proposed formation of N-CDs from arginine and ethylenediamine as precursors: (step 2a) aggregation of organic molecules (15–45 s), (step 2b) core and shell formation (30–45 s), (step 2c) shell collapse (45–75 s), and (step 2d) formation of aromatic groups within core (120–240 s). By-products are large aggregates >100 nm (steps 1a–1b) and small particles <1 kDa (step 3). Right insets: 2D excitation-emission spectra of filter residue (LA, top), dialysate (SP, middle), and carbon nanodots (CNDs) (bottom, 0.03 mg mL^−1^ in MilliQ water 25 °C), intensity is expressed in relative units. Reprinted with permission from reference [121]. Copyright © 2021, Nature Publishing Group.

The fundamental steps taking place in the bottom-up formation of CDs have been thoroughly investigated. Rigodanza et al. suggested that the formation of N-CDs from ethylenediamine and L-arginine precursors can be divided into four consecutive steps (Figure 2): (i) aggregation of small organic molecules, (ii) formation of a dense core and with an extended shell, (iii) collapse of the shell, and (iv) aromatization of the core. The findings also reveal that the N-CDs unique fluorescence is achieved already after stage (iii), when N-CDs display the final core–shell structure [121].

### 2.2. Purification of Carbon Dots

A few studies report the coexistence of different species in the final CD product [70,74,85,86]. In general, low synthesis temperature and short times tend to favor the production of fluorophores (small molecules), while high synthesis temperatures and long times are needed to form conjugated π domains (carbon cores). As a final result, both species, as well as carbon cores functionalized with fluorophores, are considered to coexist in the final product in different amounts depending on the synthetic conditions. Therefore, a variety of separation techniques have been used to purify CDs for further applications, including dialysis with membrane molecular weight cutoffs (MWCO) of 1 to 3.5 kD [37,47,122,123,124,125,126] ultrafiltration through centrifugal filter devices with different MWCO [127,128], denaturing polyacrylamide gel electrophoresis (PAGE) [129], silica gel column chromatography [49,70,74], and Sephadex column chromatography [130]. 

There are, in the literature, more extensive investigations regarding the purification of CDs. For instance, Zhang et al. gradually separated GQDs obtained from the acidic oxidation of graphene oxide using dialysis tubes with MWCO of 1, 3.5 and 7 kDa. The size of GQDs with molecular weight between 1 and 3.5, 3.5 and 7, and >7 kDa were <5 nm, 10 and 15 nm, and 16 and 20 nm, respectively, they showed different fluorescent colors and increased Quantum Yields with the size [122]. Moreover, Song et al. synthesized N-CDs from pyrolysis of citric acid and ethylenediamine, investigating the different fractions of the product via Silica gel column chromatography and via dialysis with a 3.5 kDa MWCO membrane [70]. They showed that the first fractions to leave the column were fluorophores, followed by carbon cores with attached fluorophores, and, finally, the carbon cores. When separated by dialysis, both the fraction inside the dialysis bag and that outside showed fluorescence, proving that small fluorescent molecules cross the membrane but fluorescent material (either carbon cores or carbon cores with attached fluorophores) remain in the sample. Such findings were in accordance with the work of Hinterberg et al., who synthesized N-CDs from hydrothermal pyrolysis of cysteine and citric acid and extracted the different fractions through Silica gel column chromatography [74]. Furthermore, Michaud et al. synthesized N-CDs from the pyrolysis of citric acid and cysteine and achieved a complete separation of all the components by means of two step gradient chromatography: first, silica-gel column chromatography and, second, high performance liquid chromatography (HPLC). At the end of the process, up to six components were extracted [131].

### 2.3. Metal Doping, Modification, and Post-Processing of Carbon Dots

The introduction of metals into the carbon matrix of the CDs, modifies the charge density, which, in turn, affects the physicochemical properties of CDs and can affect the catalytic properties. Hydrothermal strategies are the most common ones for the synthesis of metal-doped CDs. In such procedures, the metal precursor is mixed in solution with the organic carbon/nitrogen source and the mixture is thermally treated. Iron is the most reported dopant metal and has been introduced from different sources, such as iron benzoate [132], FeCl_3_ [133,134,135], or Fe-gluconate [136]. Cobalt has been introduced from CoCl_2_ [137] and Co-gluconate [136]. Copper has been introduced from CuCl_2_ [138,139], Cu(NO_3_)_2_ [140], Cu(Ac)_2_ [64], and CuSO_4_ [141]. Other metal sources are Zn-gluconate [142] and MnCl_2_ [143]. Finally, metalorganic complexes have also been used as carbon, nitrogen, and metal precursors at the same time. For instance, Nickel porphyrin [78] and Na_2_[Cu(EDTA)] [144] have been used to synthesize Nickel and Copper doped-CDs, respectively. 

In addition to metal atoms, the presence of surface functional groups of CDs strongly affects their properties. Amino, carboxy, and hydroxy groups are the most common ligands encountered in CDs and N-CDs, but they can also be introduced via covalent and noncovalent modification. There are reports in the literature describing the covalent modification (via amide coupling reactions, silylation, and other reactions including esterification, sulfonylation, and copolymerization) and about noncovalent modifications (π interactions, complexation/chelation, and electrostatic interactions), which accurately improve the luminescence properties and allow for novel applications, such as drug delivery, bioimaging, and ion or detection of small molecules. Thorough enumeration and explanation of these modifications and their applications is reviewed elsewhere [123].

Lastly, some applications require the combination of CDs with other materials, such as the few examples described here. For instance, Qu et al. co-crystallized CDs in a matrix of isophthalic acid or melamine, which strengthened the hydrogen bonds between CDs and the host matrix and, in turn, resulted in CDs with room temperature phosphorescence properties [145]. Sun et al. confined CDs within a SiO_2_ matrix, constructing an effective multi-confinement effect which lead to ultralong room temperature phosphorescence lifetime and exceptional stability [146]. Guo et al. embedded CDs in europium ions (Eu^3+^)-doped Metal Organic Framework (MOF) by simple stirring the preparation at room temperature for label-free ratiometric fluorescence detection of Fe^3+^ ions [147]. Sun et al. dispersed CDs into a cross-linked silica network on the surface of SiO_2_ nanoparticles to produce an efficient full-color emitting composite [148]. Zhou et al. co-crystallized CDs with cyanuric acid in order to modulate the bandgap emissions of CDs and produce highly emissive solid composite CD-based materials [149]. Li et al. combined MOFs and CDs by embedding the CDs into the MOF structure or by decorating the MOF surface with CDs in order to assess which approach is more favorable for charge separation and transfer [150]. Cheng et al. electrostatically assembled CDs with boron nitride nanosheets that exhibit thermal quenching resistance and white luminescence [151]. Finally, Jiang et al. embedded CDs in a boric acid and urea matrix using microwave heating which exhibited afterglow properties [152].

### 2.4. Synthesis of CDs-Composites for CO_2_ Reduction

Numerous catalysts reviewed in this work are made of CDs or GQDs combined with other metal or metal oxide structures. Synthetic approaches of these catalysts include the synthesis of both components separately followed by physical or chemical interaction, to in situ synthesis of both components. The most utilized components are nanostructured TiO_2_, Cu_x_O structures and C_3_N_4_ nanosheets.

**Figure 3:**
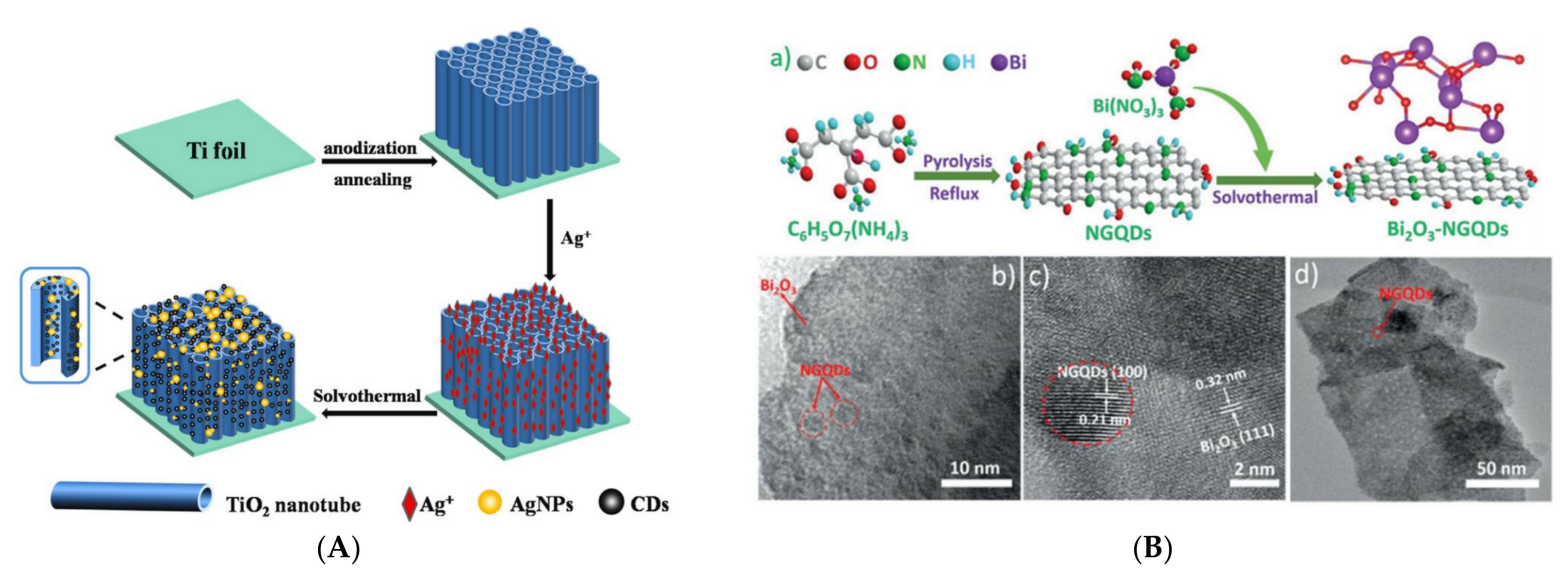
Two examples of the preparation of composite materials containing CDs or GQDs: (**A**) TiO_2_ nanotube arrays decorated with CDs and Ag NPs. Reprinted with permission from reference [83]. Copyright 2020 Elsevier Ltd. (**B**) Bi_2_O_3_ nanosheets decorated with CDs: Illustration of the preparation of Bi_2_O_3_-NGQDs (**a**), Transmission electron microscopy (TEM) (**b**,**d**) and high resolution TEM (HRTEM) (**c**) images. Reprinted with permission from reference [99]. Copyright 2018, John Wiley and sons.

CDs decoration of TiO_2_ nanoparticles (NPs) has been reported to occur by directly stirring and ultrasonicating GQDs or CDs and TiO_2_ NPs in solution [49,72]. Following another approach, Wei et al. decorated TiO_2_ nanotube arrays with both Ag NPs and CDs by a one-step solvothermal method combining the TiO_2_ nanotube arrays, AgNO_3_ and ethylene glycol as the carbon source (Figure 3A) [83]. 

A variety of copper structures have been combined with CDs. For instance, H. Li et al. supported CDs on Cu_2_O particles by in situ pyrolyzing glucose with the Cu_2_O particles [105]. In another work, Chen et al. obtained CuO-nanorods by ultrasonication of N-GQDs or N-CDs dispersed in ethanol with CuO nanorods, which were annealed at 300 °C in a second step [59]. Thirdly, Guo et al. fabricated GQDs-CuO nanocorals by simply sonicating N-GQDs with Cu_2_O nanocorals. The obtained N-GQDs-Cu_2_O nanocorals were painted on carbon paper and reduced electrochemically to GQDs-CuO nanocorals [52]. 

The synthesis of CDs-C_3_N_4_ hybrids have been studied in several reports. Song et al. and Wang et al. prepared CDs-decorated C_3_N_4_ from hydrothermally treated CDs and protonated C_3_N_4_ dispersed in water [89,103]. Q. Li et al. decorated C_3_N_4_ nanosheets with CDs by hydrothermally treating the C_3_N_4_ in an ethanol and H_2_O_2_ mixed solution. By adjusting the volume ratio of ethanol and H_2_O_2_, different quantities of CDs could be anchored [153]. CDs–C_3_N_4_ hybrids have also been combined with a third component. For instance, Zhao et al. synthesized Au-nanoparticles decorated with GQDs-C_3_N_4_ following a two-step method. GQDs-C_3_N_4_ was obtained by hydrothermally treating a mixture of GQDs with urea at 550 °C. The obtained solid was dispersed in the presence of HAuCl_4_, which was irradiated in order to obtain the final catalyst [50]. Jo et al. prepared a N-CDs/CoAl-layered double hydroxide/g-C_3_N_4_ hybrid. For such purpose, C_3_N_4_ and N-CDs were ultrasonicated in an aqueous solution containing cobalt and aluminum salts. Subsequently, urea and NH_4_F were added followed by hydrothermal treatment at 120 °C [88]. Finally, Guo et al. prepared a Co_3_O_4_-GQDs-C_3_N_4_ composite by hydrothermally treating GQDs, Co_3_O_4_ and melamine at 500 °C [51]. 

Other examples of composite materials have also been described. For instance, Gao et al. prepared CDs-covered porous Ag by directly reducing porous Ag_2_O with D-glucose in NaOH solution [106]. In a similar work, Fu et al. synthesized single-crystalline Au NPs covered with CDs, which were in situ synthesized directly from surfactants onto Au NPs [97]. As a reverse approach, Cao et al. functionalized CDs with oligomeric poly(ethylene glycol) diamine and coated the dots with gold or platinum by simple solution-phase photolysis of HAuCl_4_ or H_2_PtCl_6_, taking advantage of the fact that carbon particles acted as electron donors to reduce the metal salts [107]. Chen et al. combined Bi_2_O_3_ nanosheets and CDs by solvothermal treatment of Bismuth salt dissolved in polyol media with the presence of CDs (Figure 3B) [99]. Lv et al. obtained N-CDs-decorated MoS_2_ sheets by thermally treating exfoliated MoS_2_ with exposed active sites with DMF [154]. Xia et al. synthesized Single atom catalysts (SACs) from GQDs, which were supported on CNTs. The material was obtained by stirring metal salt (Ni, Cr, Mn, Fe, Co, Cu, or Zn) aqueous solution with GQDs and, afterwards, heating the solid at 500 °C under Ar and NH_3_ [47]. Finally, Zhong et al. embedded Ni-porphyrin based CDs into the channels of a COF by doing an in situ pyrolysis of Ni-porphyrin and glucose in the presence of the COF [78].

## 3. Photocatalytic CO_2_ Reduction 

Due to their optoelectronic characteristics, CDs can act as individual photocatalysts or as part of more complex systems as a means to increase the range of wavelength absorption, to promote the separation of the photogenerated charges or to add stability to the system (see Table 1). In comparison with other carbon-based metal-free materials, CDs are able to photocatalyze CO_2_ when certain structural conditions are present. For comparison of the activity of the metal-free carbon dots photocatalysts to other carbon-based metal free materials, we refer the readers to [155,156,157].

One of the first studies about the potential of the CDs as photocatalyst was demonstrated in 2010 by Li et al., who reported the photocatalytic activity of GQDs attached to titania (TiO_2_) or silica films. The authors monitored the degradation of methylene blue after light irradiation of the GQDs-TiO_2_ system, which was shown to be efficient [49]. Shortly after, in 2011, Cao et al. confirmed that the photoinduced charge separation in CDs could be used for photocatalysis [107] (vide infra), opening the door to new experimental observations that are described below.

### 3.1. Metal-Free Catalysts

There are several examples of surface functionalized CDs that do not contain metal or metal oxide loadings. For example, in order to increase the selectivity towards a specific compound of interest, such as HCOOH, Yadav et al. prepared a photocatalyst–biocatalyst integrated system made of GQDs functionalized with the ANP chomophore (6-amino-2-(9,10-dioxo-6-[2-[perylen-3-yl]-4,5-di-p-tolyl-4,5-dihydro-1H-imidazol-1-yl]-9,10-dihydroanthracen-2-yl)-1H-benzo[de]isoquinoline-1,3(2H)-dione) in combination with a rhodium complex ([Cp*Rh(bpy)H_2_O]^2+^ Cp* = pentamethylcyclopentadienyl, bpy = 2,2′-bipyridyl), the NAD^+^/NADH pair and formate dehydrogenase. Although the GQDs or the ANP could contribute to the obtention of HCOOH, the functionalized GQDs allowed for the increased production of HCOOH one order of magnitude higher due to the increase in the light absorption ability of the photocatalyst [160]. 

Carbon-based structures, such as graphitic carbon nitride (g-C_3_N_4_), have also been functionalized (or decorated) with CDs. Despite its excellent chemical stability and tunable band structure, the photocatalytic activity of g-C_3_N_4_ is limited by low CO_2_ adsorption capacity and high electron-hole recombination rates. Thus, the formation of composites with CDs is an efficient method to promote the separation of photoexcited charge carriers. Ong et al. reported for the first time the construction of the heterojunction photocatalyst made of CDs electrostatically attached to the surface of protonated g-C_3_N_4_. The authors observed a higher than two-fold increase in the obtention of CH_4_ and CO when the CDs were present and assigned this observation to the rapid interfacial transfer of the photogenerated electrons from the carbon nitride to the CDs that reduced the electron-hole recombination rate [103]. Qian Li et al. prepared oxygen-doped g-C_3_N_4_ materials and decorated the surface with CDs, increasing the CH_4_ production nearly 14 times in comparison with pure g-C_3_N_4_ [153]. Jo et al. prepared a more complex hybrid system integrating N-CDs, g-C_3_N_4_, and CoAl layered double hydroxides (LDH) [88]. The increased CH_4_ production is assigned to the synergistic interaction between the three components. In the particular case of the N-CDs, they contribute to light absorption, electron storage, and CO_2_ adsorption sites. In contrast to these three papers, You Wang et al. designed N-CDs able to act as hole scavengers in combination with g-C_3_N_4_ with the final objective to promote water oxidation and enhance CH_3_OH production with high selectivity [89]. The N-GQDs, with graphitic structure, were synthesized by the microwave method, and concentrated around the boundaries and edges of the C_3_N_4_ nanosheets. The composite showed active production of the six-electron product CH_3_OH and oxygen after light irradiation of CO_2_ in water, which acts as the only electron donor. Consequently, the only oxidation product is O_2_. The composite was also prepared with N-CDs prepared by the solvothermal method with amorphous structure. In this case, the N-CDs acted as electron acceptor producing only the two-electron CO as a product.

In a further work, You Wang and coworkers combined the same microwave synthesized N-CDs with a carbon nitride-like polymer (FAT) where some terminal N had been replaced by O [126]. Contrarily to g-C_3_N_4_, FAT mainly traps holes that are extracted by the N-CDs, located at the interlayers of FAT. N-CDs/FAT composite selectively reduced CO_2_ to CH_3_OH by water under neutral conditions with O_2_ as the only oxidation product. The composite’s propensity of trapping and selective extraction of holes leads to significant improvement in activity when compared to the reported N-CDs/g-C_3_N_4_ composite. For instance, no product of CO from methanol oxidation could be detected. Finally, utilization of N-CDs synthesized at different microwave powers indicated that more crystallized graphite structure is favorable to the photocatalytic performance of N-CDs.

Liu et al. addressed the comparison of photocatalytic activity between amorphous CDs and graphitized GQDs as well. The authors claimed that the presence of defined and big sp^2^ domains are responsible for the efficient separation of the photogenerated electron and hole pairs [46]. Nitrogen functionalization favored the hole scavenging, creating electron accumulation sites on the sp^2^ domains in the surface that would act as electroactive sites for the CO_2_ reduction. In addition, the surface nitrogen-containing groups induced stronger chemisorption properties of the intermediates that led to higher selectivity towards CH_4_. 

**Figure 4:**
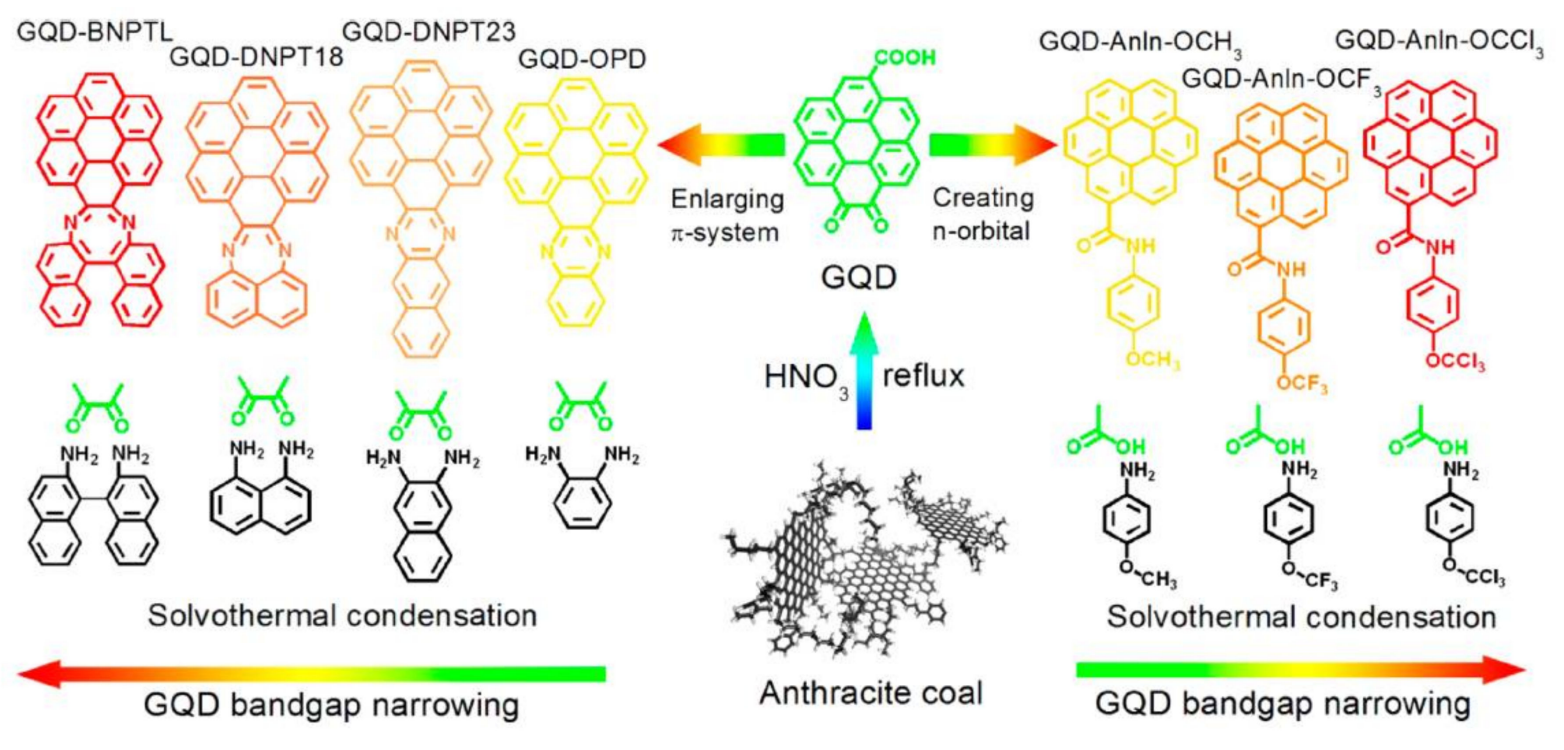
Schematic illustration of the strategies for band gap narrowing by enlarging the π-conjugated system or by introducing intermediate n-orbitals. Reprinted with permission from reference [44]. Copyright 2018 American Chemical Society.

With a similar approach, Yan et al. raised the hypothesis that the presence of intramolecular combination of p- and n-type domains separated by sp^2^-carbon ohmic contacts in GQDs favors charge separation. Moreover, if the values of the conduction band (CB) and the valence band (VB) were compatible with the standard redox potentials for water splitting or CO_2_ reduction in water, the GQDs would be able to photocatalyze these reactions. Consequently, the authors methodically tuned the band gap values of the GQDs to determine their potential activity in water splitting and CO_2_ reduction. The band gap tailoring was achieved by functionalization with polyaromatic rings to enlarge the π-conjugated sp^2^-carbon network or with electron donating groups to narrow the band gap [44] (see Figure 4). The GQDs functionalized with 1,1′-bi(2-naphthalene) (BNPTL) had the narrower band gap and the highest H_2_ yield of all the series, even higher than previously reported GQDs containing systems. The same compound also showed the highest rate for CO_2_ conversion to CH_3_OH. The enhanced light absorption, in comparison to the other molecules, and the charge separation ability were responsible for this demonstration of the GQDs potential.

### 3.2. Metal/Transition Metal-Based Hybrids and Composites

As mentioned before, Cao et al. reported in 2011 the application of gold or platinum functionalized nanoscale carbon-based structures for CO_2_ reduction in water solution, yielding HCOOH [107]. This primary study was later revisited and validated in 2014 by the same authors [161], who optimized the synthesis of the CDs and covered the surface with gold. The exposure of the system to light irradiation induced charge separation in the CDs and the subsequent electron harvesting by the gold dopant on the surface. The electron accumulation on the metal favored the creation of photocatalytic activation sites that, in combination with pressurized CO_2_ and the use of isopropanol as sacrificial electron donor, was responsible for the formation of acetic acid. It is worth mentioning that the yield of HCOOH increased along with the increasing pressure of CO_2_, suggesting the role of the CO_2_ concentration in the harvesting of photogenerated electrons in CDs. 

CDs have also been combined with other successful visible-light driven photocatalysts, such as Cu_2_O. The addition of the CDs increased the stability and the light absorption ability of the composite. In this case, the CDs acted as acceptors of the photogenerated holes and were responsible for the oxidation of H_2_O to O_2_ [105]. This approach was later enhanced by adding a carbon layer on top of the CDs to protect the semiconductor from light irradiation, increase light absorption by increasing the light reflection while acting as electron transfer layer between Cu_2_O and the CDs. As a consequence, the photocatalytic conversion of CO_2_ in water resulted in higher rates of CH_3_OH production with higher selectivity [104]. 

The combination of CDs with the popular TiO_2_ has been reported by Mengli Li et al. In the paper, the authors used CDs to expand the light absorption range of TiO_2_ which is otherwise limited to the UV part of the solar light irradiation [72]. To further enhance the photocatalytic activity of the system, the authors synthesized N,S-CDs with thiourea in order to functionalize the surface of the CDs with N, O, and S-containing groups. In the presence of CO_2_ and light irradiation, the N,S-CDs absorbed light in the visible and NIR regions and acted as electron reservoir accepting the photoinduced electrons transferred from the TiO_2_. The accumulated electrons reduced the CO_2_ molecules in solution to CO and CH_4_.

Zhong et al. reported a more complex system formed by a COF hosting a nickel porphyrin-based carbon dot (Ni-PCD@TD-COF). This was the first example in which a molecular catalyst was introduced in the porous COF network for photocatalytic CO_2_ reduction [78]. The organic framework provided higher adsorption capacity and selectivity towards CO_2_ than other semiconductors, whereas avoided the leaching of the metalloporphyrin molecule that is responsible for the catalytic activity. The CDs, in turn, provided stable active sites for the CO_2_ reduction. In combination with the photosensitizer [Ru(bpy)_3_]Cl_2_, and the triethanolamine (TEOA) which was the sacrificial reductant, the Ni-PCD@TD-COF system achieved 98% selectivity in the CO_2_-to-CO conversion over H_2_ generation.

## 4. Electrocatalytic CO_2_ Reduction 

### 4.1. Metal-Free Catalysts 

Due to their low cost, high conductivity, and surface area, non-metallic heteroatom-doped (e.g., N, S, B) carbon materials have attracted some interest as potential electrocatalysts for CO_2_RR [162,163]. Recently, polyacrylonitrile-based carbon nanofibers were found to catalyze CO_2_ conversion to CO with high faradaic efficiency (FE) in an ionic liquid electrolyte [164]. N-doped carbon nanotubes (CNTs) were also reported as effective catalysts for electrochemical CO_2_ activation and conversion in aqueous media, with preferential CO [165,166] or HCOO^−^ [167] formation apparently depending on the synthetic conditions and the chemical composition. For instance, N-doped CNT/glassy carbon (GC) electrodes obtained by ammonia plasma treatment provided a 59% FE for HCOO^−^ production in 0.1 M KHCO_3_ electrolyte, which increased to 85% upon polymer functionalization with polyethylenimine (PEI) [167]. On the other hand, CO was found to be the major CO_2_RR product formed by other N-doped CNTs synthesized using different nitrogen-containing precursors, whereby the presence of graphitic and pyridinic N defects was proposed to be key to improve CO_2_RR performances and CO selectivity [166]. In a similar fashion, N-doped graphene metal-free catalysts have been recently reported to convert CO_2_ to either CO [168,169] or HCOO^−^ [170] with high FEs, whereas boron (B)-doped graphene displayed good selectivity towards catalytic HCOO^−^ formation [171] (Table 2). Moreover, a series of metal-free carbon materials have been recently reported to efficiently catalyze CO_2_ electroreduction to further reduced products, such as acetate (CH_3_COO^−^) [172], ethanol (C_2_H_5_OH) [173,174], or methane (CH_4_) [175], in aqueous electrolytes (Table 2). 

**Figure 5:**
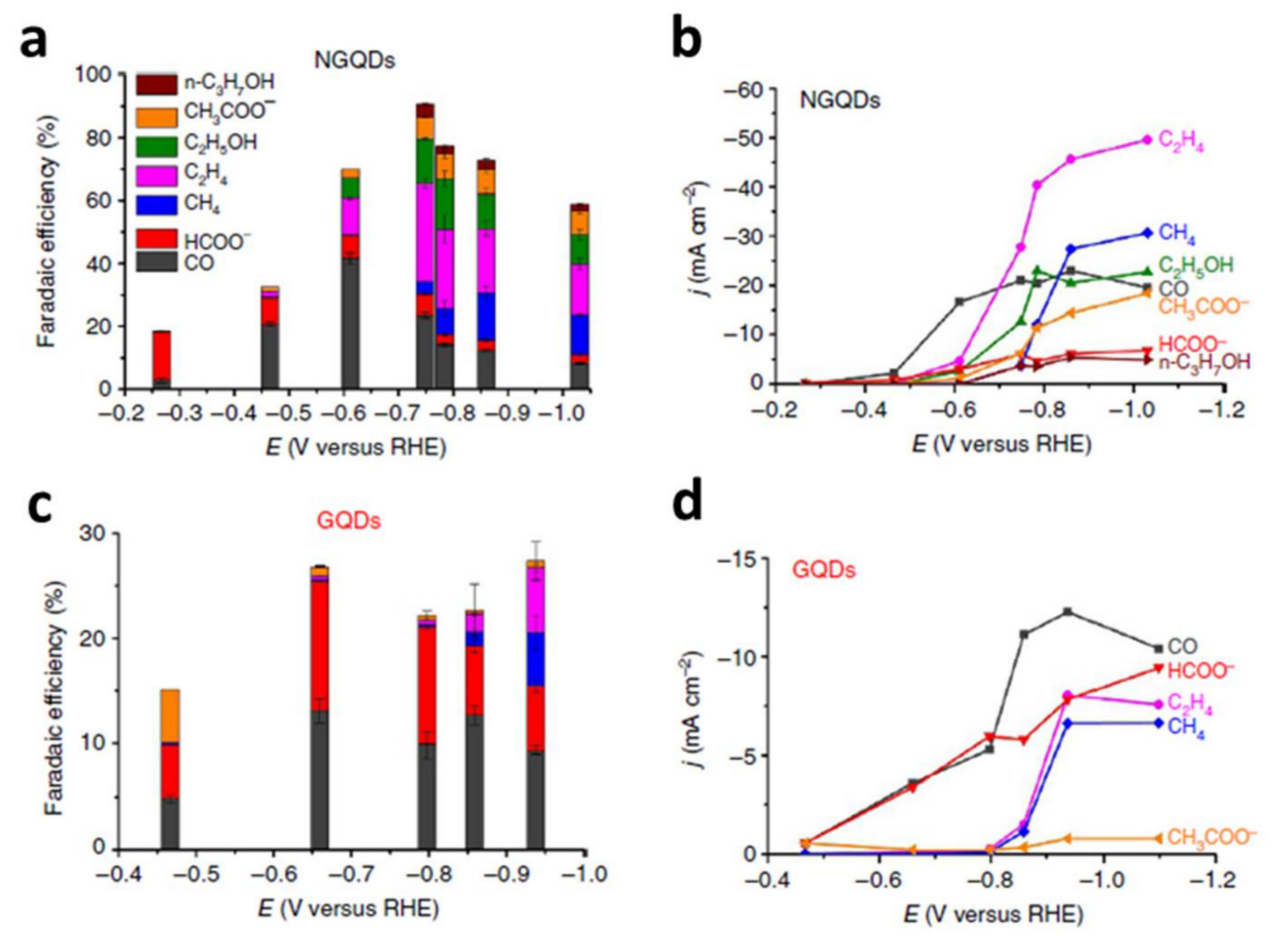
Electrocatalytic CO_2_RR activity of N-GQDs (**a**,**b**) and pristine GQDs (**c**,**d**) deposited on gas-diffusion electrodes in a flow cell in 1 M KOH electrolyte. Faradaic efficiencies (FEs, (**a**–**c**)) and partial current densities (j, (**b**–**d**)) for CO_2_ reduction toward the formation of various products (CO, CH_4_, C_2_H_4_, HCOO^−^, C_2_H_5_OH, CH_3_COO^−^, and *n*-C_3_H_7_OH) at various applied cathodic potentials. Adapted with permission from reference [17]. Copyright 2016, Nature Publishing Group.

By combining heteroatom N-doping and high density of reactive edge sites, N-GQDs featured unique electrocatalytic CO_2_RR properties. In a flow cell reactor and alkaline electrolyte (1 M KOH), N-GQDs deposited on a gas diffusion layer (GDL) electrode displayed a primary conversion of CO_2_ into CO and HCOO^−^ at relatively low overpotentials, while promoting the formation of multi-electron CO_2_RR products at more cathodic potentials, including CH_4_ (maximum FE = 15% at –0.86 V vs. RHE), as well as multi-carbon (C_≥2_) hydrocarbons and oxygenates (Table 2) [17]. In particular, C_2_H_4_ was found to be the major hydrocarbon product with a maximum FE of 31% at –0.75 V vs. RHE, whereas a maximum overall FE = 26% was obtained for multi-carbon oxygenates at –0.78 V vs. RHE (C_2_H_5_OH resulting the major component, FE_C2H5OH_ = 16%), thus providing comparable results to previously reported Cu-based nanostructured catalysts (Figure 5 a,b) [17,176]. In comparison with N-GQDs, pristine GQDs only produced CO/HCOO^−^ mixtures with lower FEs together with only traces of CH_4_/C_2_H_4_ and no detectable amounts of oxygenates, suggesting the critical role of nitrogen moieties incorporated into the conjugated carbon nanostructure (Figure 5c,d). Furthermore, noticeable amounts of hydrocarbons and oxygenates were also detected on control micro-sized N-doped reduced graphene oxide (N-RGO), albeit at a considerably higher overpotential than N-GQDs, thus highlighting the importance of the nanoscale morphology for the optimization of the CO_2_RR performances. It was proposed that the enriched pyridinic N doping at the exposed edge sites is at the origin of the outstanding activity and selectivity towards C_≥2_ hydrocarbons and oxygenates observed for N-GQDs [17,176]. In a follow-up work, first-principles calculations were used to provide theoretical insights into the possible pathways for CO_2_RR catalyzed by N-GQDs and to rationalize the experimentally observed selectivity [177]. The *COOH binding step (* indicates the adsorption site), crucial for the initial two-electron reduction of CO_2_ to CO, was predicted to be energetically more favorable in the presence of N-doped edges compared to N-doped graphene. Moreover, the preferential formation of CH_4_ over CH_3_OH was proposed to originate from the decreased kinetic barrier for the step leading to the key *CH_2_ intermediate from *CH_2_OH assisted by H_2_O molecules, which enable a more favorable H shuttling pathway for the dissociation process [177]. On the other hand, the formation of C_≥2_ hydrocarbons and oxygenates is primarily determined by the subsequent coupling of *CH_2_ and CO to form the *CH_2_CO intermediate, engaging the pyridine N site and the nearest-neighbor zig-zag edge C atom [177], in sharp contrast with the main pathway commonly reported for C–C bond formation by Cu catalysts which involves CO dimerization step [178]. 

Surface functionalization was recently revealed to be an effective strategy to tune the catalytic activity and selectivity of GQDs towards the formation of specific CO_2_RR products, by altering the electronic structure of the catalyst, as well as the adsorption energy of key surface-bound intermediates. In particular, GQDs featuring varying contents of specific functional groups on their surface were systematically investigated for CO_2_RR, allowing to correlate the catalytic performances with the electronic properties of the substituents [100]. As a general trend, the presence of electron-withdrawing groups onto GQDs (e.g., -COOH, -SO_3_) tend to suppress CO_2_RR favoring HER. Contrariwise, GQDs decorated with electron-donating functional groups (e.g., –OH, -NH_2_) were found to enhance CO_2_RR selectivity towards CH_4_ formation. Such a boosting effect was found to be even more pronounced for GQDs enriched with NH_2_ functionalities, leading to maximum FE and partial current density of 70.0% and −200 mA cm^−2^ for CH_4_ production, respectively [100]. In agreement with these findings, an in situ amine functionalization strategy of N-CDs which ensured a better control over the total and the specific N active sites incorporated into the carbon nanostructure, led to a significant improvement of the CO_2_-to-CH_4_ performances [90]. The total N atomic content in the N-GQDs was found to positively impact the overall CO_2_RR performances, whereas the catalytic selectivity was dependent on the specific type of N configurations. The FE and production rate for CH_4_ and C_2_ products displayed a linear correlation with the content of –NH_2_ and pyridinic N dopants, respectively, whereas any specific relationships with CO_2_RR selectivity toward a certain product were obtained for pyrrolic N, graphitic N, and nitrogen oxide functionalities [90]. 

### 4.2. Transition Metal-Based Single-Atom Catalysts

**Figure 6:**
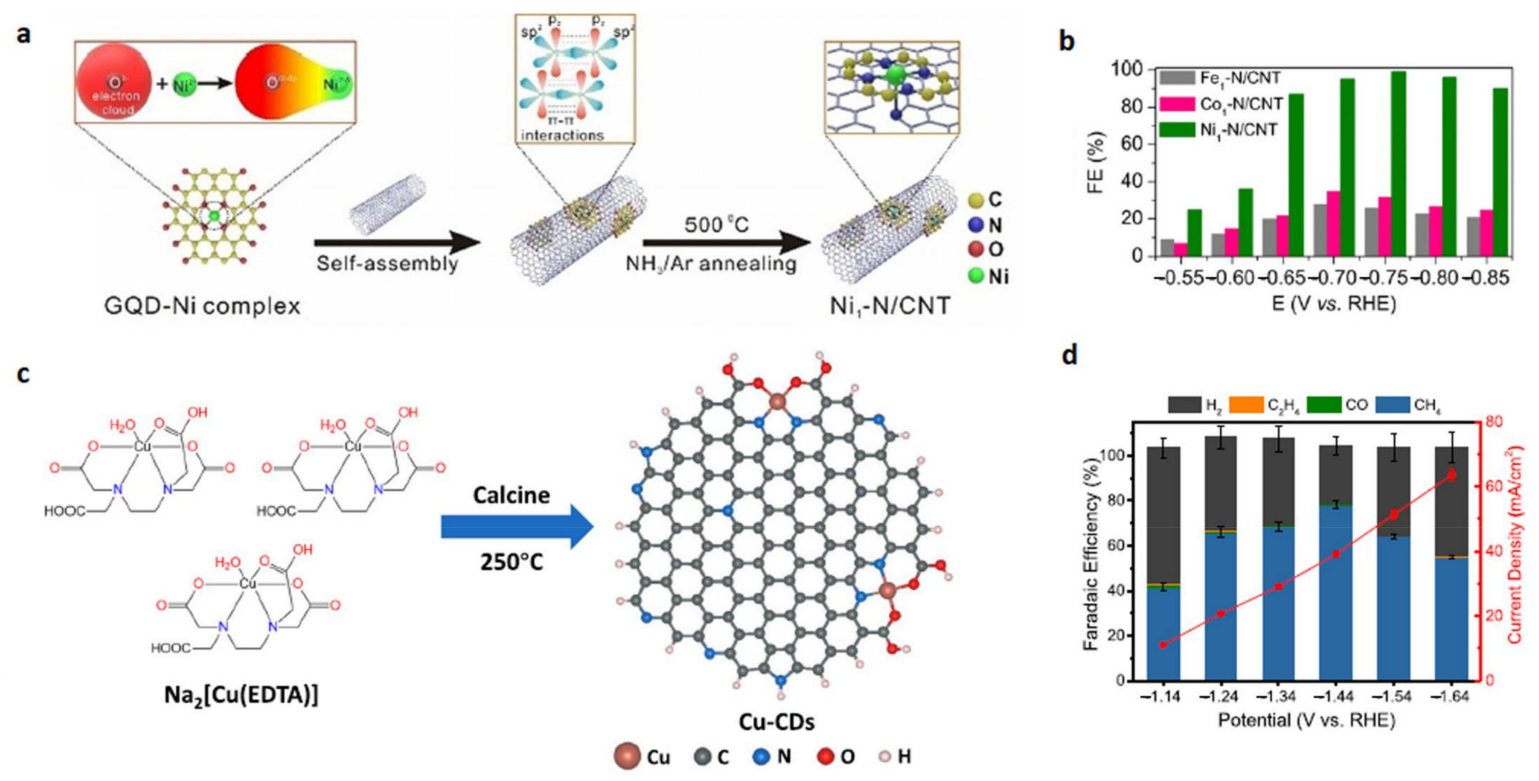
(**a**,**b**) Illustration of the preparation of M_1_-N/CNT SACs (M = Fe, Co, Ni) (**a**) and their observed FEs for CO_2_RR to CO at various applied potentials (**b**). Adapted with permission from reference [47]. Copyright 2020, Wiley-VCH. (**c**,**d**) Scheme of the low-temperature calcining procedure for Cu-CDs catalyst (**c**) and dependence of FE (left y-axis) and current density (based on geometric surface area, right y-axis) of Cu-CDs (**d**). Adapted with permission from reference [144] Copyright 2021, Nature Publishing Group.

In the last years, metal-doped graphene quantum dots (M-GQDs) have been explored as suitable precursors to obtain atomically dispersed transition metal-based single atom catalysts (SACs) for an efficient and selective CO_2_RR in aqueous electrolytes [47,101]. For instance, a versatile GQD-tethering strategy for SACs synthesis was recently developed by Jin et al., consisting in an initial metal ion complexation into a GQD to form a stable coordination M-GQD adduct, which is then assembled on CNT support via π–π interactions (Figure 6a) [47]. Ultimately, the hybrid M-GQD/CNT material undergoes high-temperature annealing under NH_3_/Ar leading to uniformly distributed M-N_x_ moieties on a conductive carbon support. In comparison with the traditional top–down and bottom–up methods for SACs synthesis [179], this approach takes advantage of the stable metal-GQD interaction which prevents agglomeration of metal atoms during the pyrolysis treatment. Furthermore, the abundance of oxygenated functional groups and defective sites on the surface of GQDs increases their capability of anchoring metal ions, resulting in SACs with considerably higher metal loadings compared to those obtained by more conventional methods. Following this approach, a series of SACs containing different transition metal atoms (including Cr, Mn, Fe, Co, Ni, Cu, Zn) with metal loading between 3.0 and 4.5 % wt. were successfully synthesized and tested for CO_2_RR in 0.1 M KHCO_3_ electrolyte [47]. Among them, the Ni_1_-N/CNT catalyst exhibited excellent selectivity and durability for CO_2_-to-CO conversion, achieving a maximum FE_CO_ = 99% at –0.75 V vs. RHE (Figure 6b) and maintaining an almost quantitative FE for CO production over 60 h operation [47]. More recently, an analogous synthetic method based on the use of strongly chelating amine-functionalized GQDs as precursors for metal atom trapping enabled to obtain SACs with different transition metals possessing further increased metal loadings up to a record value of ca. 40% wt. [101]. For example, the atomically dispersed Ni-N-C-3 catalyst displayed a metal loading of approximately 15% wt., considerably higher than the values commonly reported for Ni SACs [180,181], providing outstanding CO_2_RR performances for selective CO production (>90% selectivity and a ca. 122 mA cm^−2^ partial current density) at a cell voltage of ca. 2.55 V in a flow electrochemical cell [101].

In some cases, high annealing temperatures are not required to obtain carbon dots-based SACs for efficient CO_2_RR. Recently, mild-temperature calcination of the organometallic Na_2_[Cu(EDTA)] complex ultimately led to the formation of a CDs-supported SAC containing well-defined CuN_2_O_2_ moieties, reminiscent of the original metal atom coordinative sites of the molecular precursor (Figure 6c) [144]. The resulting Cu-CDs material displayed unique catalytic CO_2_RR properties, with extremely high FEs (maximum FE_CH4_ ~ 78% at −1.44 V vs. RHE) and selectivity toward CH_4_ formation (>99% of the CO_2_RR products) (Figure 6d), outperforming the reference CuPc catalysts in terms either CH_4_ selectivity and catalyst stability. Both experimental and theoretical investigation highlighted the pivotal role of the CuN_2_O_2_ sites on the unusually high observed catalytic CH_4_ selectivity [144]. 

### 4.3. Metal/Metal-Oxide Composites 

**Figure 7:**
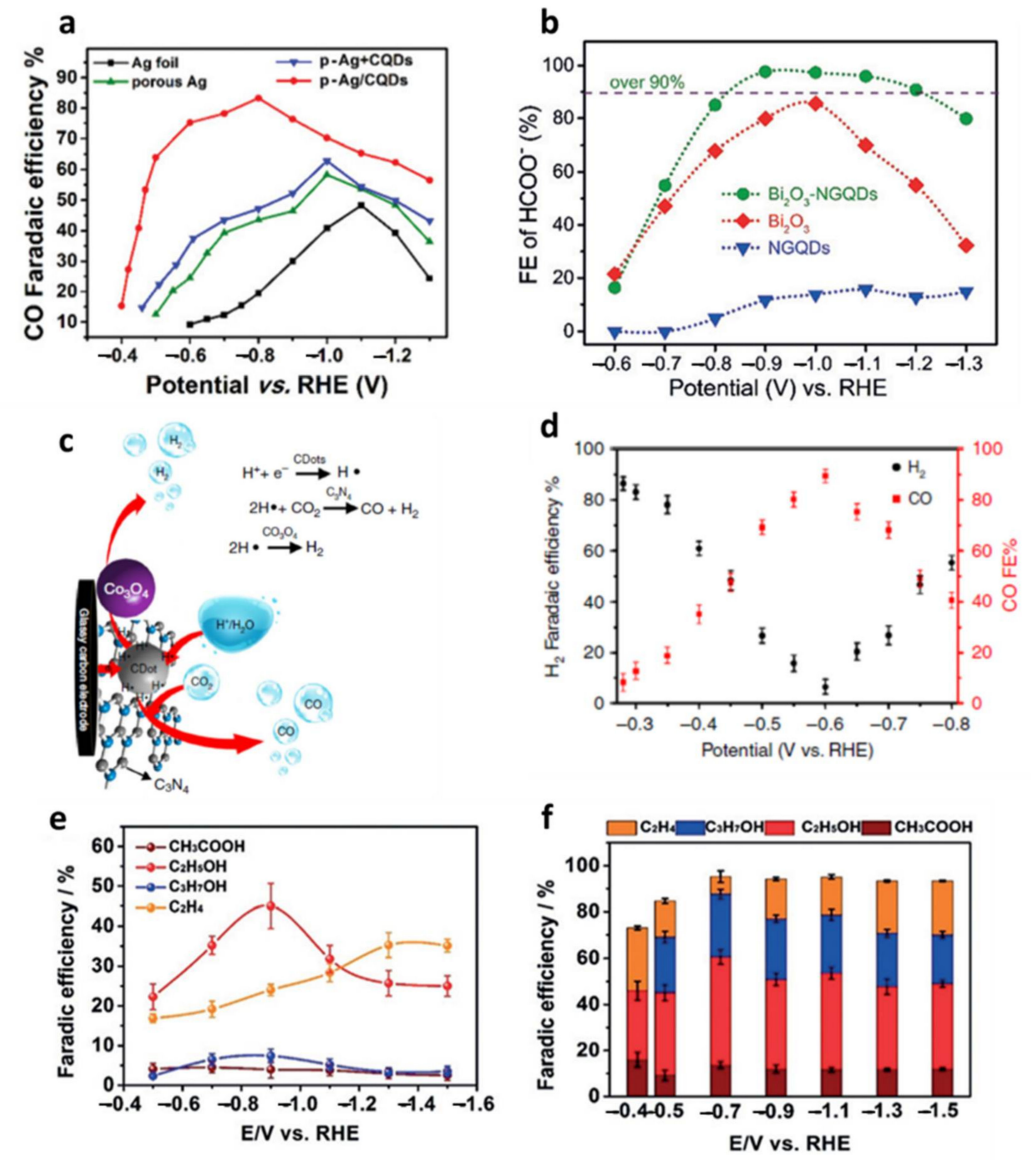
(**a**) FE_CO_ vs. the applied potential of CD-covered porous Ag (p-Ag/CDs) in CO_2_-saturated 0.5 M KHCO_3_ electrolyte. Adapted with permission from reference [106]. Copyright 2019, Royal Society of Chemistry. (**b**) FE of HCOO^−^ production on Bi_2_O_3_-N-GQDs, Bi_2_O_3_ and N-GQDs at different electrolytic potentials. Adapted with permission from reference [99]. Copyright 2018, Wiley-VCH. (**c**) Schematic diagram of the reaction mechanism induced by Co_3_O_4_-CDs-C_3_N_4_. CDs are the generation site of H^•^, Co_3_O_4_ the generation site of H_2_, and C_3_N_4_ the generation site of CO. (**d**) FEs of the reduction of CO_2_ to CO (red points) and H^+^ to H_2_ (black points) catalyzed by Co_3_O_4_-CDs-C_3_N_4_ vs. the applied potential. Experiments were performed in triplicates and results are shown as mean ± standard deviation. Adapted with permission from reference [51]. Copyright 2017, Nature Publishing Group. (**e**,**f**) The distribution of C_≥2_ products at different potentials over N-GQD/Cu-nr during CO_2_RR and CORR experiments. Adapted with permission from reference [59]. Copyright 2020, Wiley-VCH.

CDs can be also used in combination with catalytically active metal or metal-oxide nanostructured materials, leading to hybrid catalysts with improved electrocatalytic CO_2_RR performances. In these composite catalysts, the presence of CDs may enable the tuning of CO_2_RR selectivity toward specific products or boost their intrinsic activity, acting as a co-catalyst component or providing additional catalytic active sites. Two-electron CO_2_ reduction products, such as CO and formate, are the most commonly products formed by metallic heterogeneous catalysts, although selectivity is still a major challenge, especially in aqueous electrolytes. Noble metals, such as Ag [182,183,184] and Au [185,186,187], are the most widely studied transition metals for selective CO production due to their favorable energies for CO desorption [188,189], whereas a few examples of other metals, such as Pb [190], Sn [191], and Bi [192], have been reported to catalyze HCOO^−^ production with relatively high faradaic yields. In general, the hybridization of nanostructured metal catalysts with CDs has been demonstrated to be beneficial for the CO_2_RR catalytic performances. For instance, a porous Ag/CDs composite catalyst was found to selectively reduce CO_2_ to CO with a maximum FE of 83.2% at −0.8 V vs. RHE, outperforming both the control polycrystalline Ag foil and the CD-free porous Ag samples in terms of onset potential required for CO_2_ conversion to CO and CO selectivity (Figure 7a) [106]. Although the CDs alone displayed predominant HER activity, they were found to positively impact the capability of CO_2_ adsorption and CO desorption on porous Ag, leading to the experimentally observed catalytic improvement of CO_2_RR-to-CO properties of the material. In a similar fashion, single-crystalline Au NPs wrapped by N-CDs served as highly active and CO-selective electrocatalyst, being capable of catalytically converting CO_2_ to CO at a considerably lower overpotential compared to the CDs-free Au NPs, as well as sustaining high FE_CO_ values in a wide potential range (−0.25 V and −0.65 V vs. RHE) [97]. It was proposed that a synergistic effect between the two components is responsible for an improved *COOH binding on pyridinic N sites of the composite catalyst, which ultimately results in enhanced CO_2_RR performances. Following an analogous approach, the combination of nanostructured Bi_2_O_3_ or SnO_2_ materials and N-CDs led to highly active composite catalysts for selective HCOO^−^ formation in aqueous electrolyte (FE> 90% between −0.9 V and −1.2 V vs. RHE for Bi_2_O_3_/N-CDs catalyst) (Figure 7b) [99].

Furthermore, the strategy of CDs hybridization was successfully applied to boost the electrocatalytic CO_2_RR properties of transition metal chalcogenides, usually affected by limited CO_2_RR selectivity and poor electronic conductivity [193]. In particular, a MoS_2_/N-CDs nanocomposite material with high N-doping content, exhibited a superior activity compared to bare exfoliated MoS_2_ in CO_2_-saturated EMIM-BF_4_ solutions (94 mol% water), showing significantly improved rates, overpotential, and selectivity toward CO formation (FE = 90.2% at −0.9 V vs. RHE) [154]. The origin of the observed boosting effect was ascribed to the presence of N-CDs, which contribute not only to increase the conductivity of the material but also to lower the barrier for rate-limiting *COOH binding step. 

In addition to binary composites based on the combination of CDs and a metal/metal-oxide catalyst, Guo et al. recently developed the novel versatile design concept of a three-component hybrid catalyst for electrochemically-driven syngas production from simultaneous CO_2_RR and HER in aqueous electrolyte [51]. As depicted in Figure 7c, the system comprises: (i) an earth-abundant transition metal-based HER catalyst, e.g., Co_3_O_4_; (ii) porous graphitic C_3_N_4_, accounting for CO_2_ conversion to CO due to the presence of N dopants and ensuring well NPs dispersion; (iii) CDs, acting as the generation site for active hydrogen (H^•^) by trapping H^+^ and e^−^, thus triggering both the reaction channels. In a CO_2_-saturated aqueous electrolyte at a close-to-neutral pH, the multi-component catalyst was found to start catalyzing CO_2_RR to CO at −0.28 V vs. RHE, corresponding to a very low overpotential (0.17 V), and afforded highly stable syngas production over 100 h electrolysis test [51]. Importantly, the H_2_:CO ratio could be fine-tuned from 0.07:1 to 4:1 by controlling the applied potential (Figure 7d) or, alternatively, the amount of Co_3_O_4_ HER catalyst. A series of dedicated experiments unraveled the specific role played by each component within the composite, highlighting the improved H^•^ stabilization and CO_2_/H^+^ adsorption promoted by CDs as critical steps for CO production by C_3_N_4_-CDs, notwithstanding the CDs alone could only produce H_2_. Analogous results were reported for a ternary composite based on a combination of Au NPs and CDs loaded on C_3_N_4_, which was recently found as a highly stable electrocatalyst for CO_2_ reduction to CO [50]. Under optimal conditions, the latter achieved a sustained CO production with a maximum FE of ca. 79.8% at −0.5 V vs. RHE, outperforming the bare Au NPs and the other combinatorial two-component catalysts. The superior CO_2_RR performances of the ternary Au-CDs-C_3_N_4_ composite suggest the presence of a synergistic effect among all the three components. In particular, CDs were supposed to play a major role on the enhanced catalytic response, due to their capability of CO_2_/H^+^ adsorption and increased conductivity [50]. 

In addition to CO and HCOO^−^, multi-electron conversion of CO_2_ into value-added chemicals, such as oxygenates and multi-carbon hydrocarbons (C_≥2_), is highly desirable. Copper-based electrocatalysts are, so far, the most promising transition metal-based systems for converting CO_2_ into target C_≥2_ products [194,195,196,197,198,199]. Although electrochemically-driven C-C coupling process efficiently occurs on Cu catalysts, usually leading to predominant ethylene formation [200,201], CO_2_RR selectivity toward the formation a specific product is generally low. Recently, the presence of CDs in composite Cu nanocatalysts has been reported to induce a drastic change in selectivity for CO_2_RR. For instance, hybrid Cu-CDs nanocorals were found to catalyze HCOO^−^ formation at low overpotentials (0.13 V), leading to a maximum 68% faradaic yield at −0.7 V vs. RHE, with a minor amount of detected CH_3_OH accounting for ca. 11% FE [52]. In contrast, CDs-free Cu nanocorals mainly promoted HER under the same conditions, thus indicating the crucial role CDs in catalyzing CO_2_RR over HER. More recently, Chen et al. reported a two-component catalyst based on CuO-derived Cu nanorods and N-GQDs (N-GQD/Cu-nr), capable of efficiently converting CO_2_ to multi-carbon alcohols with outstanding high efficiencies [59]. In a flow cell reactor and an alkaline aqueous electrolyte (1 M KOH), NGQ/Cu-nr was found to produce C_≥2_ products with FE up to 80.4% (corresponding to a 282.1 mA cm^−2^ current density) at −0.9 V vs. RHE, of which a 52.4% FE accounting for C_≥2_ alcohols (Figure 7e), thus favorably comparing to the state-of-the-art Cu catalysts. More specifically, the presence of N-GQDs, alone capable of catalyzing CO_2_RR to CO and C_2_ products, significantly contributed to shift selectivity toward the alcohol formation, as indicated by the increase in the alcohol/ethylene ratio from 0.81 on Cu-nr to 2.18 on N-GQD/Cu-nr. Notably, the N-GQD/Cu-nr composite catalyst displayed also excellent performances for electrocatalytic CO reduction reaction (CORR), resulting in an overall FE for C_≥2_ alcohols formation up to 75.3% at −0.7 V vs. RHE with a partial FE of 27.2% for n-propanol formation and an over 10-fold higher alcohol-to-ethylene ratio compared to Cu-nr (Figure 7f) [59]. The preferential production of C_≥2_ alcohols on N-GQD/Cu-nr was mainly ascribed to a synergistic cooperation effect between the Cu-based nanorods and N-GQDs, both acting as active sites for C_≥2_ formation. In particular, DFT calculations suggested that the increase oxophilicity due to the incorporation of N-GQDs induced an enhanced stabilization of the oxygenic key *CH_2_CHO intermediate, thus favoring the formation of alcohols through further carbon protonation [59].

## 5. Summary and Future Outlook

In this review, we have surveyed the utilization of carbon dots for photocatalytic and electrocatalytic conversion of CO_2_ to C_1_ and C_≥2_ products. Due to their unique optical and electrical properties, the CDs are able to photocatalyze the reduction of CO_2_ alone or in combination with other components as part of more elaborated catalytic systems. In photocatalytic CO_2_RR, the CDs or GQDs act as electron acceptors and strongly contribute to enhance light absorption, while providing active sites for the catalytic reaction. GQDs were also found to be active materials for electrocatalytic CO_2_RR, being able to catalyze the process themselves or playing a synergistic role in combination with other components. As metal-free catalysts, GQDs were reported to promote multi-electron CO_2_ conversion in aqueous electrolytes, leading to the formation of C_1_ or C_≥2_ hydrocarbons and oxygenates with efficiencies depending on the applied potential. N-doping and the specific configuration of N sites present in the material are critical for CO_2_RR selectivity and activity of GQDs. The incorporation of pyridinic N dopants was proposed to provide suitable active sites for catalytic formation of C_≥2_ hydrocarbons and oxygenates via an alternative mechanism to that commonly proposed for Cu-based electrocatalysts. On the other hand, when implemented into binary or ternary metal/metal-oxide composite materials, the GQDs displayed beneficial effects on the catalytic activity of the nanostructured catalysts, improving the overall CO_2_RR performances in terms of selectivity, activity, and overpotential. In hybrid catalysts, the functionalization with GQDs was proposed to alter the adsorption energies of key intermediates, display synergistic effects, favor CO_2_ uptake and improve conductivity, resulting in optimal CO_2_RR catalytic properties. Furthermore, due to the unique intrinsic physicochemical features of GQDs, metal ion complexation with GQDs was recently proposed as an effective strategy for the synthesis of single-atom catalysts for efficient and selective CO_2_RR, preventing metal–metal agglomeration during the pyrolysis step and leading to metal loadings considerably higher than those reported by conventional approaches. 

Due to the extreme sensitivity of both photocatalytic and electrocatalytic CO_2_RR performances to the structure and surface composition of carbon dots, future research on the synthesis and characterization of the carbon nanoparticles is required in order to control the composition and its influence on the redox properties. The development of well-defined reproducible protocols for their synthesis and purification is highly desired to improve the rational design of CD-based catalysts. At the same time, the systematic characterization of CD-based materials by using ex situ and operando/in situ spectroscopic techniques is required to establish a direct correlation between structural features and catalytic properties. Operando/in situ spectroscopy also provides a useful experimental tool to get more detailed structural information about the key intermediates involved in the process and the active sites, as well as to real-time monitor their structural evolution under catalytic conditions. This would also improve the rational understanding of the fundamental role played by the CDs in the catalytic reaction, which is an essential step for further improvement of the field. Moreover, the investigation of model systems based on the functionalization of molecular catalysts with CDs would also contribute to unravel the influence of CDs on the photochemical, redox, and electronic properties of the catalysts at a fundamental level. We are confident that the overview of the literature data herein presented will contribute to shed light on the role played by the CDs in the electrochemical and photochemical CO_2_RR, helping to direct future research in the field.

## Figures and Tables

**Table 1:** Description of the photocatalytic systems containing carbon dots for CO_2_ reduction. Two carbon-based materials have also been added as reference.

Catalyst	Major Products	Yield	Reaction Conditions
g-C_3_N_4_/carbon nanosheets [158]	CO, CH_4_	229 µmol CO/g 112 µmol CH_4_/g (in 7 h)	H_2_O
Graphene Oxide [159]	CH_3_OH	0.172 µmol/g·h	H_2_O
Ni-PCD@TD-COF ^1^ [78]	CO, H_2_	956 µmol CO/g (in 2 h) 19 µmol H_2_/g (in 2 h)	ACN:H_2_O:TEOA 3:1:1 ^2^
CDs-CN ^3^ [89]	CH_3_OH, CO	13.9 µmol CH_3_OH/g·h 0.05 µmol CO/g·h	H_2_O
CDs-FAT ^4^ [126]	CH_3_OH	24.2 µmol CH_3_OH/g·h	H_2_O
CDs-g-C_3_N_4_ 3% wt. [103]	CH_4_, CO	37.06 µmol CH_4_/g 68.8 µmol CO/g (in 10 h)	H_2_O
CL@CDs/Cu_2_O [104]	CH_4_, CH_3_OH	99.6 µmol CH_3_OH/g·h 8 µmol CH_4_/g·h	H_2_O, TEOA
CDs/Cu_2_O [105]	CH_3_OH	55.7 µmol CH_3_OH/g·h	H_2_O and dry ice
N-CDs/LDH/g-C_3_N_4_ [88]	CH_4_	25.4 µmol CH_4_/g·h	H_2_O
CDs/oxygen doped C_3_N_4_ [153]	CH_4_	1.2 µmol CH_4_/g·(in 8 h)	H_2_O
GQDs-BNPTL [44]	CH_3_OH	0.695 µmol CH_3_OH/g·h	H_2_O
GQDs vs CDs [46]	CDs: CO	400 µmol CO/g·h,	H_2_O
	GQDs: CH_4_	983 µmol CH_4_/g·h,	
N,S-CDs/TiO_2_ [72]	CO, CH_4_	1.838 µmol CO 1.195 µmol CH_4_ (in 6 h)	H_2_O
GQDs-ANP ^5^/biocatalyst [160]	HCOOH	198 µmol HCOOH (in 2 h)	H_2_O, TEOA
CDs@PEG-Au [161]	HCOOH CH_3_COOH	1.2 µmol HCOOH/g·h 0.06 µmol CH_3_COOH/g·h	H_2_O

^1 ^Ni-PCD: Ni(II) porphyrin attached to a Carbon Dot, confined in the channels of the TD functionalized COF. Ru sensitizer: [Ru(bpy)_3_]Cl_2._^2^ ACN: acetonitrile, TEOA: triethanolamine. ^3^ CN: carbon nanosheets. ^4^ FAT: polymer-like carbon nitride. ^5^ ANP: 6-amino-2-(9,10-dioxo-6-(erylenelen-3-yl)-4,5-di-p-tolyl-4,5-dihydro-1H-im- idazol-1-yl)-9,10-dihydroanthracen-2-yl)-1H- benzo[de]isoquino- line-1,3(2H)-dione.

**Table 2:** Electrocatalytic CO_2_RR performances reported for selected systems based on carbon dots and on other metal-free carbon materials.

Catalyst	Electrolyte	Major Product(s) (FE, %)	Applied Potential (vs. RHE)
N-doped 3D graphene foam [169]	0.1 M KHCO_3_	CO (85%)	−0.58 V
N-doped graphene [170]	0.5 M KHCO_3_	HCOO^−^ (73%)	−0.84 V
N-doped nanodiamond/Si rod array (NDD/Si RA) [172]	0.5 M NaHCO_3_	CH_3_COO^−^ (77.3−77.6%)	−0.8 V/−1.0 V
Perfluorinated covalent triazine framework (CTF) [175]	0.1 M KHCO_3_	CH_4_ (99.3%)	−0.7 V/−0.9 V
N-doped cylindrical mesoporous carbon [173]	0.1 M KHCO_3_	CH_3_CH_2_OH (77%)	−0.56 V
B-/N-co-doped nanodiamond [174]	0.1 M NaHCO_3_	CH_3_CH_2_OH (93.2%)	−1.0 V
N-GQDs [17]	1 M KOH	CH_4_ (15%)	–0.86 V
		C_2_H_4_ (31%)	–0.75 V
NH_2_-functionalized GQDs [100]	1 M KOH	CH_4_ (70.0%)	−0.95 V
Ni_1_-N/CNT [47]	0.1 M KHCO_3_	CO (99%)	–0.75 V
Cu-CDs [144]	0.5 M KHCO_3_	CH_4_ (78%)	−1.44 V
porous Ag/CDs [106]	0.5 M KHCO_3_	CO (83.2%)	−0.8 V
Au NPs/N-GQDs [97]	0.5 M KHCO_3_	CO (93%)	−0.25 V
Bi_2_O_3_-N-GQDs [99]	0.5 M KHCO_3_	HCOO^−^ (98.1%)	−0.9 V
MoS_2_/N-CDs [154]	EMIM-BF_4_ (94 mol% water) ^1^	CO (90.2%)	−0.9 V
Co_3_O_4_-CDs-C_3_N_4_ [51]	0.5 M KHCO_3_	CO (89%)	−0.6 V
Au-CDs-C_3_N_4_ [50]	0.5 M KHCO_3_	CO (79.8%)	−0.5 V
Cu-GQDs nanocorals [52]	0.5 M KHCO_3_	HCOO^−^ (68%) CH_3_OH (11%)	−0.7 V
N-GQDs/Cu-nr [59]	1 M KOH	C_≥2_ products (80.4%) C_≥2_ alcohols (52.4%)	−0.9 V

^1 ^EMIM-BF_4_ = 1-Ethyl-3-methylimidazolium Tetrafluoroborate.

## Data Availability

Not applicable.
